# c-Abl regulates gastrointestinal muscularis propria homeostasis via ERKs

**DOI:** 10.1038/s41598-017-03569-0

**Published:** 2017-06-15

**Authors:** Jinnan Xiang, Yiqun Zhang, Dandan Bao, Na Cao, Xin Zhang, Ping Li, Shoutao Qiu, Jigang Guo, Dan He, Baojie Li, Liqing Yao, Huijuan Liu

**Affiliations:** 1 Bio-X Institutes, Key Laboratory for the Genetics of Developmental and Neuropsychiatric Disorders, Ministry of Education, Shanghai Jiao Tong University, Shanghai, 200240 China; 2 Endoscopy Center and Endoscopy Research Institute, Zhongshan Hospital, Fudan University, 180 Fenglin Road, Shanghai, 200032 China

## Abstract

The gastrointestinal tract is responsible for food digestion and absorption. The muscularis propria propels the foodstuff through the GI tract and defects in intestine motility may cause obstruction disorders. Our present genetic studies identified non-receptor tyrosine kinase c-Abl as an important regulator of the muscularis propria homeostasis and a risk factor for rectal prolapse. Mouse deficient for *c*-*Abl* showed defects in the muscularis propria of gastrointestinal tract and older *c*-*Abl* ^−/−^ mice developed megaesophagus and rectal prolapse. Inhibition of c-Abl with imatinib mesylate, an anti-CML drug, or ablation of c-Abl using Prx1-Cre, which marks smooth muscle cells, recapitulated most of the muscularis propria phenotypes. The pathogenesis of rectal prolapse was attributable to overproliferation of smooth muscle cells, which was caused by enhanced ERK1/2 activation. Administration of ERK inhibitor U0126 impeded the development of rectal prolapse in c-Abl deficient mice. These results reveal a role for c-Abl-regulated smooth muscle proliferation in the pathogenesis of rectal prolapse, and imply that long-term use of imatinib mesylate may cause gastrointestinal problems in patients while ERK inhibitor may be effective in treating rectal prolapse.

## Introduction

The gastrointestinal (GI) tract is responsible for food digestion, nutrient absorption, and defecation^1, 2^, which has four concentric layers: mucosa, submucosa, muscularis propria, and adventitia or serosa. The mucosa is composed of epithelium, lamina propria, and muscularis mucosae, which are highly specialized in different parts of the GI tract to support its local functions. The submucosa consists of a dense irregular layer of connective tissue with blood and lymphatic vessels. The muscularis propria consists of an inner circular layer and an outer longitudinal layer. The outermost layer consists of several layers of connective tissue^3^. The muscularis propria is in charge of transporting foodstuff along the gastrointestinal tract. Defects in muscularis propria, due to myocyte degeneration and/or fibrosis or structural abnormalities of the tissue^4^, may cause intestine motility problems and intestine obstruction^5, 6^. Yet, the etiology of the GI motility disorders remains unclear.

*c*-*Abl* is a proto-oncogene that encodes abelson tyrosine kinase^7–9^. Its oncogenic form, BCR-ABL, regarded as the causes of the chronic myeloid leukemia (CML) development^10^. The development of imatinib mesylate (Gleevec, STI571) as an inhibitor of Abl kinases and a potent drug for treatment of CML, gastrointestinal stromal tumors (GISTs), and a number of other cancers, is a great success in the management of cancer^11–13^. Despite extensive studies of BCR-ABL and CML, the physiological function of c-Abl is not fully understood^9^. c-Abl can be activated by growth factor, cell adhesion, and stress especially genotoxic stress and oxidative stress^8, 9, 14, 15^. c-Abl null mice showed perinatal lethality, runtedness, lymphopenia, reduced fertility, osteoporosis, and heart problems^16–18^. These phenotypes imply that c-Abl may play a role in aging^19–22^. In addition, a role for c-Abl in the immune cells and neuronal cells has been well established^9, 23–25^. However, the molecular mechanisms by which c-Abl deficiency results in other defects in mice remain unclear.

Here, we showed that *c*-*Abl* ^−/−^ mice or normal mice treated with imatinib mesylate showed altered homeostasis of the GI tract muscularis propria, manifested by thicker muscle layers and myocyte disorganization. Moreover, *c*-*Abl* ^−/−^ mice gradually developed megaesophagus and rectal prolapse. Ablation of c-Abl in smooth muscle cells recapitulated most of the muscularis propria phenotypes, suggesting a smooth muscle cell-autonomous effect for c-Abl in causing these defects. The altered homeostasis of the muscularis propria and development of prolapse were shown to be caused by increased proliferation of smooth muscle cells due to enhanced ERK1/2 activation. These findings indicate that c-Abl is an important regulator of muscularis propria homeostasis and a potential disease gene for rectal prolapse, and suggest that pathogenesis of rectal prolapse may involve overproliferation of smooth muscle cells. The clinical implications of our study are that imatinib mesylate may affect the homeostasis of the GI tract in CML patients and that ERK inhibitors may be effective in treating rectal prolapse.

## Results

### *c*-*Abl*^−/−^ mice showed defects in esophagus muscularis propria and developed megaesophagus

**Figure 1:**
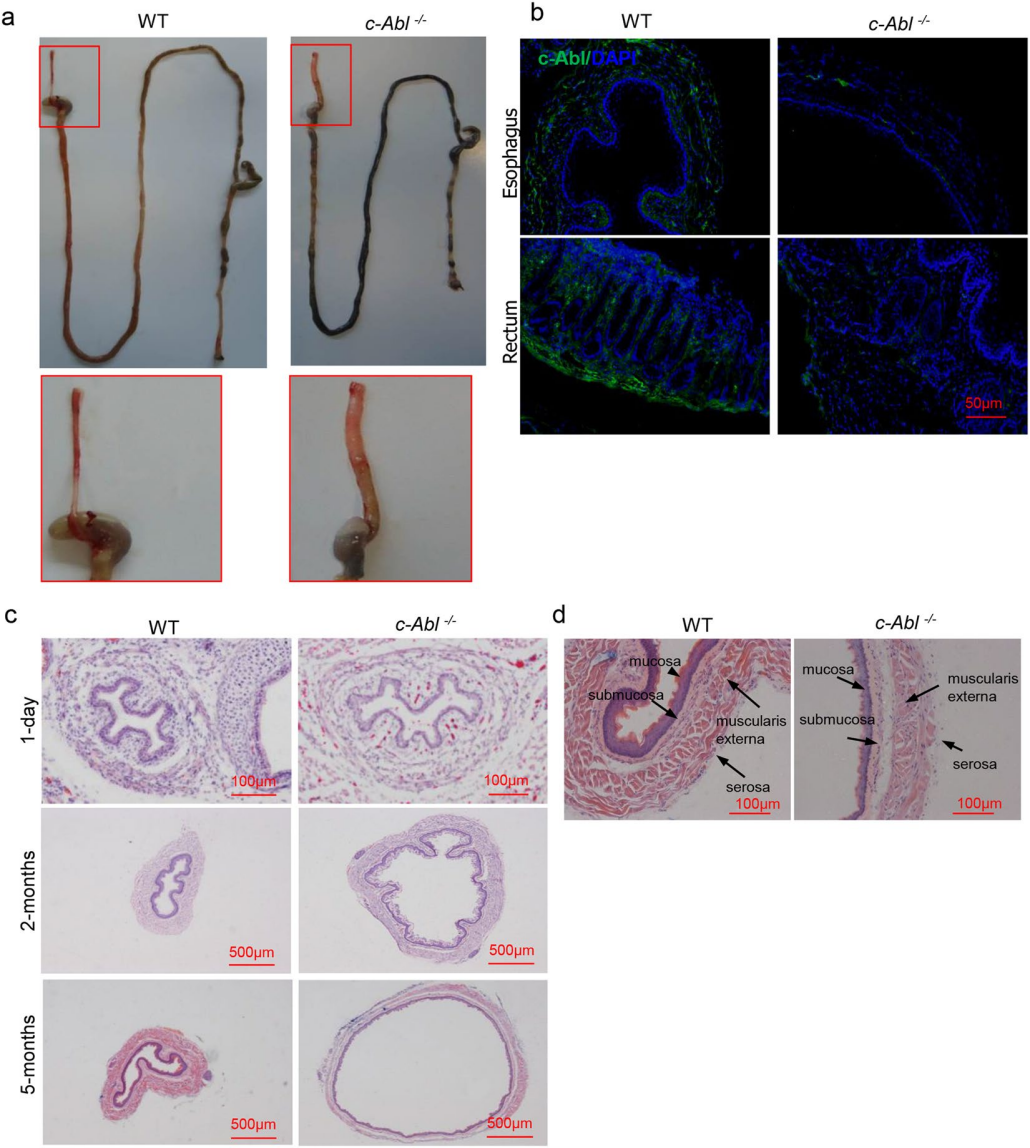
*c*-*Abl* ^−/−^ mice showed progressive development of megaesophagus phenotype. (**a**) The whole GI tracts of wild type and *c*-*Abl* ^−/−^ mice. The GI tracts were dissected out and rinsed with cold PBS, from which the pictures were taken. N = 5. (**b**) c-Abl expression was detectable in the submucosa and muscular externa but not epithelial cells of esophagus and colorectal tissues. N = 3. (**c**) Representative histological sections of esophagus of day 1, 2 month, and 5 month-old *c*-*Abl* ^−/−^ and wild type mice. The organs were paraffin-embedded and the section slides were stained with hematoxylin and eosin. N = 3. (**d**) High magnification of muscles in the esophagus of 5 month-old *c*-*Abl* ^−/−^ and wild type mice. The organs were paraffin-embedded and the section slides were stained with hematoxylin and eosin. N = 3.

To investigate the role of c-Abl in the GI tract, we dissected out the whole GI tracts from 5-month-old *c*-*Abl* ^−/−^ and wild type littermates, and found that *c*-*Abl* ^−/−^ mice had enlarged but shortened esophagus as well as a swollen anus, which were not observed in mice deficient for Arg (an c-Abl paralog) (Fig. 1a and data not shown). Consistent with previous studies, c-Abl is expressed in the whole GI tract^26^, we found that c-Abl protein was detectable in esophagus and colorectal tissues, mainly in the submucosa and muscular externa but not in epithelial cells (Fig. 1b). The dilation of the esophagus was associated with peristalsis problems as food was often seen stuck in the middle of the esophagus. However, the esophagus-stomach junction of *c*-*Abl* ^−/−^ mice did not show the “bird’s beak” morphology characteristic of achalasia (Fig. 1a and Supplementary Fig. S1)^27^.

We then sectioned the esophagus of *c*-*Abl* ^−/−^ and wild type mice of various ages and stained them with hematoxylin and eosin (Fig. 1c). All the six *c*-*Abl* ^−/−^ mice older than 2 months showed an increase in the diameter, although to various degrees. This phenotype was not obvious in newborn or 1-month old *c*-*Abl* ^−/−^ mice (Fig. 1c and data not shown). Under higher magnifications, while the circular and the longitudinal muscle layers were tidily organized and well separated from each other in normal esophagus, the muscle fibers were not properly aligned or separated into distinct layers in *c*-*Abl* ^−/−^ mice. In older mice, the muscularis propria layer is thinner (Fig. 1c and d), although the cross-section area of the muscle fibers even showed a modest increase (1.43 ± 0.35 fold, p < 0.05) and the total number of smooth muscle cells was increased by 1.0 fold in *c*-*Abl* ^−/−^ mouse esophagus (Supplementary Fig. S2). The morphology of the epithelium layer also looked slightly altered (Fig. 1d), which might be secondary to muscularis propria defects as c-Abl is not expressed in epithelial cells (Fig. 1b). In sum, *c*-*Abl* ^−/−^ mice presents a megaesophagus model that is likely caused by muscularis propria defects.

### *c*-*Abl*^−/−^ mice showed anomalies in the muscularis propria of stomach, colon and rectum and developed rectal prolapse

We then compared other parts of the GI tracts of *c*-*Abl* ^−/−^ and wild type mice and observed an obvious alteration in the forestomach of *c*-*Abl* ^−/−^ mice, with an increase in the thickness of the muscle layers and the secretory mucosa (Supplementary Fig. S3a). However, in glandular stomach and intestine, the difference is minimal (Supplementary Fig. S3b). The number and the size of the intestine villi were not altered by c-Abl deficiency, nor was the muscle layer, which is rather thin in the intestine (Supplementary Fig. S3c). The colon showed a thicker muscle layer in *c*-*Abl* ^−/−^ mice (Supplementary Fig. S3d). The differential effects of c-Abl deficiency on esophagus, stomach, and intestines may be caused by the different nature of the muscle tissues in these organs. However, the distribution of food, chime, and metabolic waste along the digestive tract looked normal in the mutant mice (Supplementary Fig. S1), suggesting that the gut motility was not impaired by c-Abl ablation. Mouse esophagus muscularis propria contains both skeletal muscle and smooth muscle while stomach and intestines muscularis propria is composed of solely smooth muscle.

**Figure 2:**
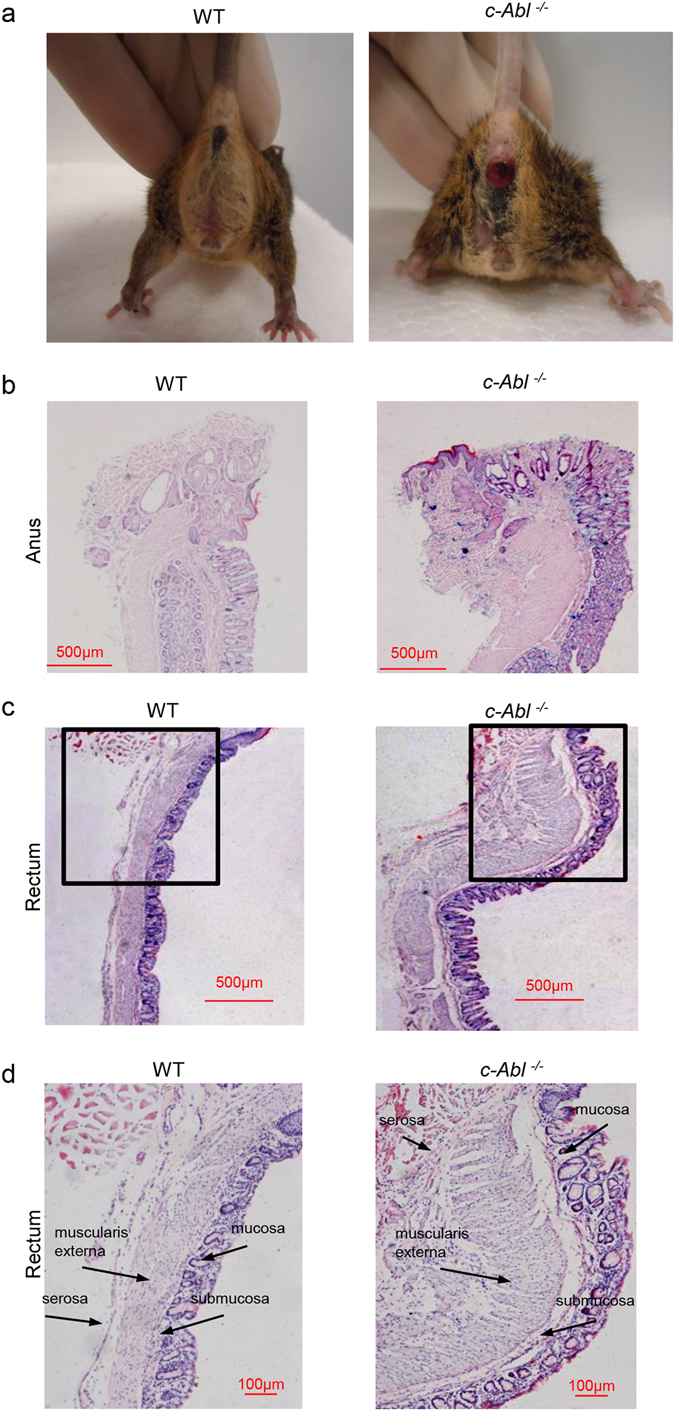
*c*-*Abl* ^−/−^ mice showed muscularis propria defects and rectal prolapse. (**a**) Representative picture of rectal prolapse in *c*-*Abl* ^−/−^ mice. N = 5. (**b**) Representative histological sections of the anus of *c*-*Abl* ^−/−^ and wild type mice. The organs were paraffin-embedded and the section slides were stained with Hematoxylin and eosin. N = 5. (**c**) Representative histological sections of the colon-rectum junction of *c*-*Abl* ^−/−^ and wild type mice. The organs were paraffin-embedded and the section slides were stained with Hematoxylin and eosin. N = 5. (**d**) Higher magnification of Fig. 2c. N = 5.

Another obvious problem of *c*-*Abl* ^−/−^ mouse is rectal prolapse. While 2 month-old *c*-*Abl* ^−/−^ mice seldom showed rectal prolapse, all 4–5 month-old-mice showed this phenotype (Fig. 2a). In human, rectal prolapse is a disorder that affects mainly elderly women and its etiology is still unknown^28^. It is estimated that the annual incidence of rectal prolapse is 2.5 per 100, 000 people^29, 30^. Histological analysis showed that *c*-*Abl* ^−/−^ mice had an increase in the thickness of the smooth muscle layer and the number of smooth muscle cells in the rectal prolapse tissues (Fig. 2b and c). The muscle layers were also thicker in the colon-rectum junction region of c-Abl deficient mice (Fig. 2d). Thus, *c*-*Abl* ^−/−^ mice showed a thickened muscularis propria in the stomach, colon, and rectum and represents a model of atypical rectal prolapse.

### Imatinib mesylate treatment also resulted in muscularis propria defects in mouse GI tract

**Figure 3:**
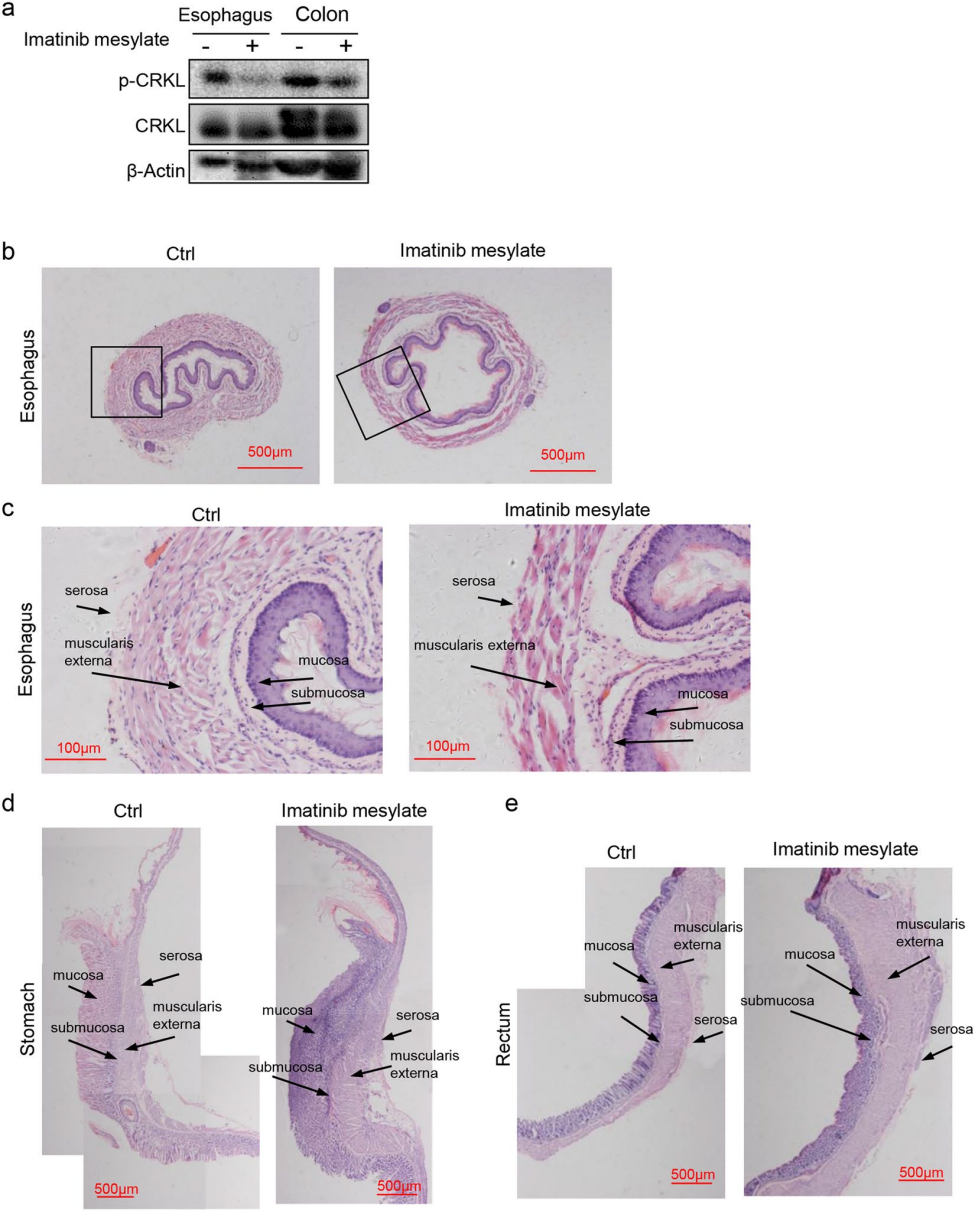
Imatinib mesylate administration led to defects in muscularis propria of the GI tract. (**a**) Administration of imatinib mesylate inhibited c-Abl activation in esophagus and colon tissues, manifested by a decrease in the phosphorylation of c-CRKL, a phosphorylation substrate of Abl kinases. N = 3. (**b**) Representative histological sections of the esophagus in imatinib mesylate-treated and control mice. The organs were paraffin-embedded and the section slides were stained with Hematoxylin and eosin. N = 3. (**c**) High magnification: representative histological sections of the esophagus in imatinib mesylate-treated and control mice. The organs were paraffin-embedded and the section slides were stained with Hematoxylin and eosin. N = 3. (**d**) Representative histological sections of the stomach in imatinib mesylate-treated and control mice. The organs were paraffin-embedded and the section slides were stained with Hematoxylin and eosin. N = 3. (**e**) Representative histological sections of the rectum in imatinib mesylate-treated and control mice. The organs were paraffin-embedded and the section slides were stained with Hematoxylin and eosin. N = 3.

We then determined whether long-term administration of imatinib mesylate showed any effects on the GI tract in mice, with comparison to *c*-*Abl* ^−/−^ mice. Two-month-old normal mice were injected with 50 mg/kg imatinib mesylate every other day for 14 weeks. This dose was shown to inhibit c-Abl activation in esophagus and rectal tissues, manifested by a decrease in the levels of phosphorylation of CRKL, a substrate of Abl kinases (Fig. 3a, Supplementary Fig. S4 and data not shown). We found that compared to control mice that received saline injection, imatinib mesylate led to a slight reduction of body weight in mice (data not shown). However, imatinib mesylate seemed not to cause obvious morphological change in the whole GI tract (data not shown).

Careful histological analysis of the esophagus, stomach, and rectum revealed that imatinib mesylate treatment showed an effect on the muscularis propria of all three organs. In esophagus, the muscularis propria showed a thinner muscle layer and distorted muscle fibers alignment (Fig. 3b and c), which were similar to *c*-*Abl* ^−/−^ mice (Fig. 1c and d). On the other hand, the forestomach and the large intestines showed an increase in the thickness of the muscularis propria in imatinib mesylate-treated mice (Fig. 3d and e), which were in general consistent with the GI alteration observed in *c*-*Abl* ^−/−^ mice. However, imatinib mesylate administration showed no further effect on the phenotypes of *c*-*Abl* ^−/−^ mice (data not shown), suggesting there is no additive effect between c-Abl deficiency and inhibition.

Unlike *c*-*Abl* ^−/−^ mice, mice receiving imatinib mesylate treatment did not develop megaesophagus or rectal prolapse. The reason can be that c-Abl plays a role in the early development, c-Abl has kinase-independent functions, c-Abl is not completely inhibited by 50 mg/kg imatinib mesylate administrated every 48 hours, or the combination of these possibilities.

### Ablation of c-Abl in smooth muscle cells led to muscularis propria defects and development of rectal prolapse

**Figure 4:**
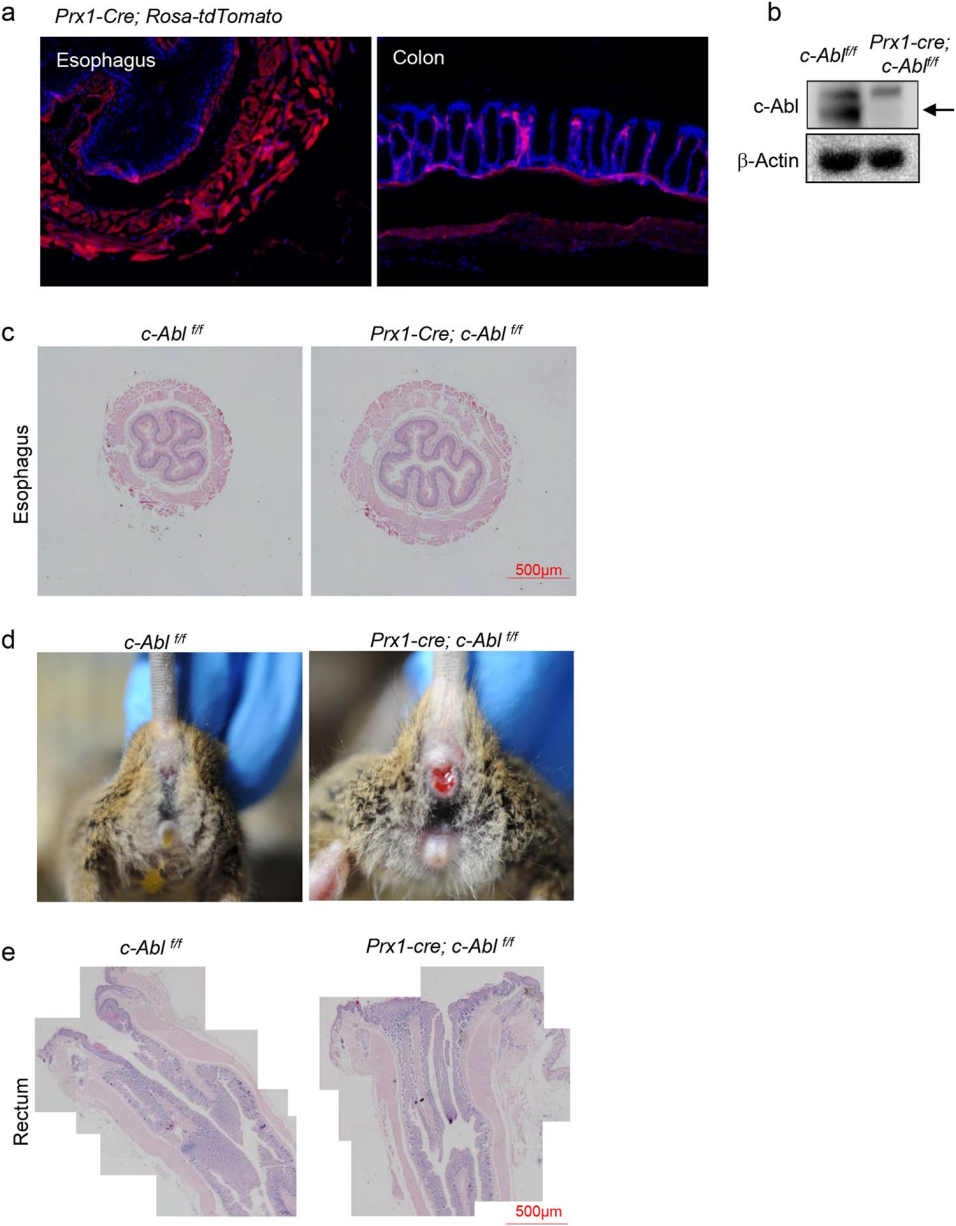
*Prx1*-*Cre*; *c*-*Abl* ^*f/f*^ mice showed muscularis propria defects and rectal prolapse. (**a**) Lineage tracing experiment show that the esophagus and colorectal muscularis propria were labeled by Prx1. The organs were dissected from *Prx1*-*Cre*; *Rosa*-*tdTomato* mice, frozen sectioned and observed under fluorescence microscope. N = 3. (**b**) Western blot results showed that the expression of c-Abl is diminished in *Prx1*-*Cre*; *c*-*Abl* ^*f/f*^ mouse rectum smooth muscle tissues. N = 3. (**c**) Representative histological sections of the esophagus of *Prx1*-*Cre*; *c*-*Abl* ^*f/f*^ and control mice. The organs were paraffin-embedded and the section slides were stained with Hematoxylin and eosin. N = 3. (**d**) Representative images of the rectal prolapse in *Prx1*-*Cre*; *c*-*Abl* ^*f/f*^ mice. N = 3. (**e**) Representative histological sections of the colon-rectum junction of *Prx1*-*Cre*; *c*-*Abl* ^*f/f*^ and wild type mice. The organs were paraffin-embedded and the section slides were stained with Hematoxylin and eosin. N = 3.

The above findings suggest that c-Abl may play critical roles in GI muscularis propria homeostasis and c-Abl deficiency leads to the development of megaesophagus and rectal prolapse. To test whether c-Abl plays a cell-autonomous role in smooth muscle cells, we crossed *c*-*Abl* ^*f/f*^ mice to *Prx1*-*Cre* mice, which is a marker for mesenchymal stem cells (MSCs)^31^. It is well established MSCs can differentiate into smooth muscle cells in addition to osteoblasts and chondrocytes^32, 33^. Lineage tracing experiment using *Prx1*-*Cre*; *Rosa*-*tdTomato* mice revealed that Prx1 labeled the smooth muscle cells of esophagus and colon muscularis propria (Fig. 4a). *Prx1*-*Cre*; *c*-*Abl* ^*f/f*^ mice showed a decrease in the protein level of c-Abl in the colorectal smooth muscle tissues (Fig. 4b and Supplementary Fig. S5). These mutant mice developed prolapse and a modest megaesophagus (Fig. 4c and d). The rectum of *Prx1*-*Cre*; *c*-*Abl* ^*f/f*^ mice also showed increased thickness of smooth muscle layer and the number of smooth muscle cells (Fig. 4e and data not shown). These results suggest that c-Abl regulates the homeostasis of muscularis propria in a smooth muscle cell-autonomous manner.

### A role for p16INK4a in the development of rectal prolapse

The above studies revealed a role for c-Abl in the development of megaesophagus and rectal prolapse in older mice. *c*-*Abl* ^−/−^ mice are known to develop other aging-related phenotypes including lymphopenia, senile osteoporosis, and shortened lifespan^16–18^. One of the important regulators of aging is p16INK4a, a CDK inhibitor that is up-regulated in senescent cells and tissues^34, 35^. We then generated *c*-*Abl* ^−/−^ *p16Ink4a* ^−/−^ mice and we found that *p16Ink4a* deficiency partially rescued the neonatal lethality of *c*-*Abl* ^−/−^ mice (Supplementary results Fig. S6a), as well as the reduction in fertility, without affecting the body weight and the organ weight of liver, spleen, kidney, or testes (Supplementary results Fig. S6b,c and data not shown)^36^. While none of the 6 *c*-*Abl* ^−/−^ male mice tested was fertile, all 6 *c*-*Abl* ^−/−^ *p16Ink4a* ^−/−^ mice could give rise to offspring when crossed to wild type female mice^37^. However, p16INK4a deficiency failed to rescue the megaesophagus phenotype of *c*-*Abl* ^−/−^ mice and only slightly rescued the rectal prolapse phenotype of *c*-*Abl* ^−/−^ mice (Supplementary results Fig. S6d and e). These results, taken together, suggest that c-Abl deficiency-induced onset of megaesophagus and rectal prolapse is not likely to be mediated by the pro-aging protein p16INK4a.

### c-Abl deficiency or inhibition promoted smooth muscle cell proliferation

We found that the rectal smooth muscle layer was thickened and the number of smooth muscle cells in the esophagus was also increased in *c*-*Abl* ^−/−^ mouse (Supplementary Fig. S2), suggesting c-Abl deficiency caused overgrowth of smooth muscle in these two tissues. On the other hand, the structure of the skeletal muscle in the esophagus of *c*-*Abl* ^−/−^ mice appeared normal (Fig. 1d). Staining for ganglia in the esophagus and rectum revealed no alteration in *c*-*Abl* deficient mice (Supplementary Fig. S7). Moreover, the colorectal crypts showed normal cell proliferation (Supplementary Fig. S8a). Lastly, c-Abl deficiency did not cause infiltration of CD3^+^ cells into the esophagus or rectum (Supplementary Fig. S9), nor did it affect the expression of inflammatory cytokines, e.g., TNFα, MPO, and IL6, in the intestines (data not shown), excluding possible involvement of inflammation in the development of the atypical rectal lapse. These results, together with the findings that ablation of c-Abl in Prx1+ mesenchymal cells reproduced the esophagus and rectal phenotypes, suggest that megaesophagus and rectal prolapse may be caused by defects in smooth muscle cells.

**Figure 5:**
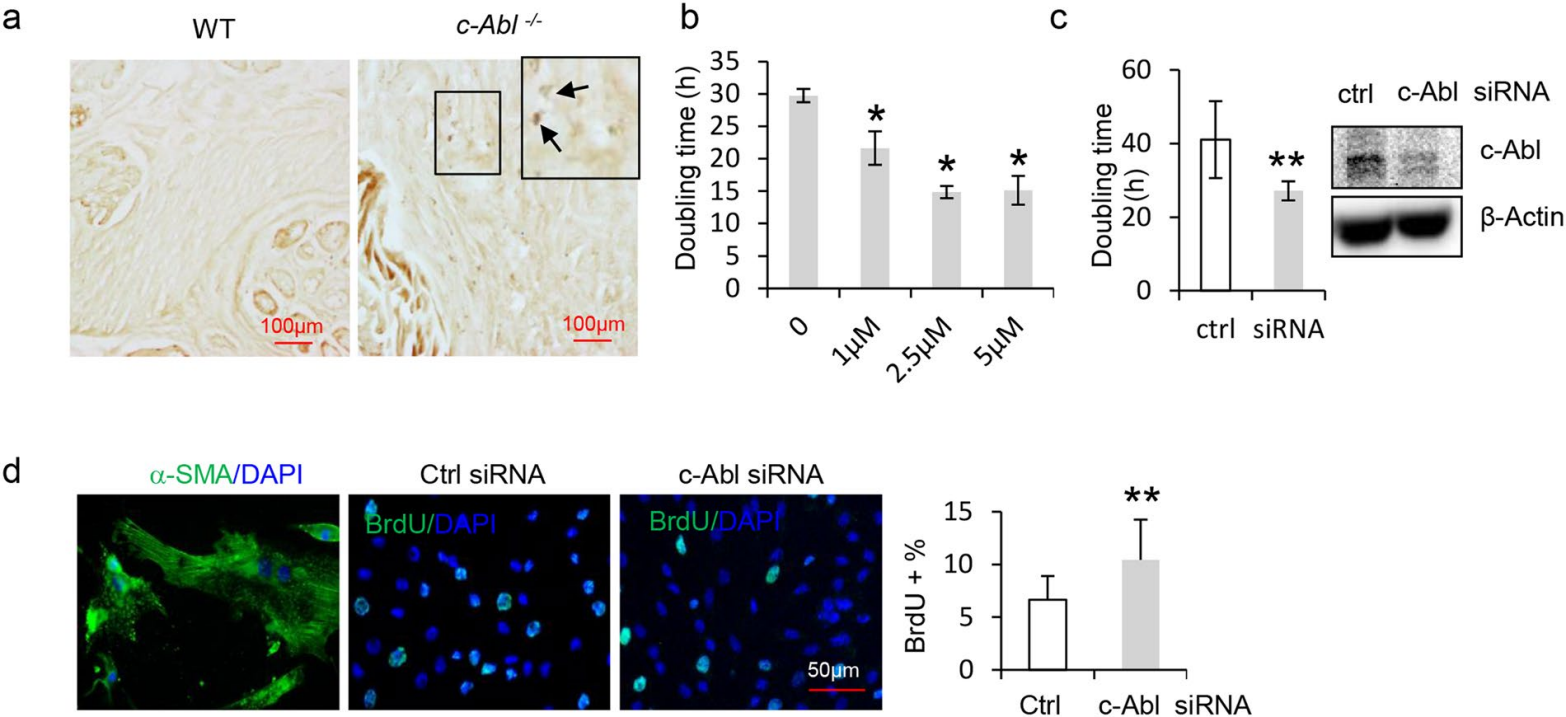
Inhibition or ablation of c-Abl resulted in enhanced proliferation of smooth muscle cells. (**a**) *c*-*Abl* ^−/−^ mouse rectum showed an increase in the number of Ki67 positive cells compared to WT mouse. N = 3. (**b**) Imatinib mesylate treatment led to a decrease in the doubling time in smooth muscle cells. p = 0.027 (1 μM), 0.021 (2.5 μM), 0.010 (5 μM), *p < 0.05 when compared to control. N = 3. (**c**) Knockdown of c-Abl led to a decrease in the doubling time in smooth muscle cells. Right panel: siRNA knockdown of c-Abl. p = 0.005, **p < 0.01 when compared to control siRNA. N = 5. (**d**) c-Abl knockdown in primary esophagus smooth muscle cells led to an increase in cell proliferation, judged by an increase in the BrdU-labeled S phase cells. The left panel showed that the cells expressed smooth muscle marker αSMA. For c-Abl knockdown, see Fig. 5c. p = 0.003, **p < 0.01. N = 5.

We found that *c*-*Abl* deficient mouse rectum showed an increased number of Ki67-positive cells (Fig. 5a). Yet the esophagus showed an insignificant change in cells positive for Ki67 (Supplementary Fig. S8b). This could be due to slow progress of megaesophagus (a 1-fold-increase in the number of smooth muscle cells over a period of 5 months). These results suggest that that c-Abl deficiency results in overproliferation of smooth muscle cells. Previous studies have shown that knockdown of c-Abl affects myoblast and smooth muscle cell adhesion and proliferation *in vitro* ^38–41^. We then tested whether imatinib mesylate show any effects on the proliferation, cell death, and morphology of smooth muscle cells. It was found that imatinib mesylate led to a modest but significant increase in the proliferation rates of smooth muscle cells, but not primary skeletal muscle cells (Fig. 5b and Supplementary Fig. S8c). Moreover, imatinib mesylate treatment showed no effect on death rates or morphology of all three cell types (data not shown).

Moreover, siRNA-mediated c-Abl knockdown led to accelerated proliferation of αSMA-expressing primary esophagus smooth muscle cells, manifested by a decrease in doubling time and an increase in BrdU-labeled S phase cells, without affecting the cell cycle profiles (Fig. 5c,d, Supplementary Fig. S10, and data not shown). c-Abl deficiency also led to an increase in the number of proliferating cells in primary rectal smooth muscle cells (Supplementary Fig. S8f). c-Abl knockdown smooth muscle cells also showed an increase in proliferation in response to PDGF-AA (Supplementary Fig. S8e). This is not consistent with a previous report showing that c-Abl is required for PDGF-AA or ET-1-induced proliferation in rat vascular smooth muscle cells^38^. This discrepancy may be caused by differences in cell types and/or cell culture conditions and warrants further investigation.

### c-Abl deficiency increased smooth muscle cell proliferation via ERK

**Figure 6:**
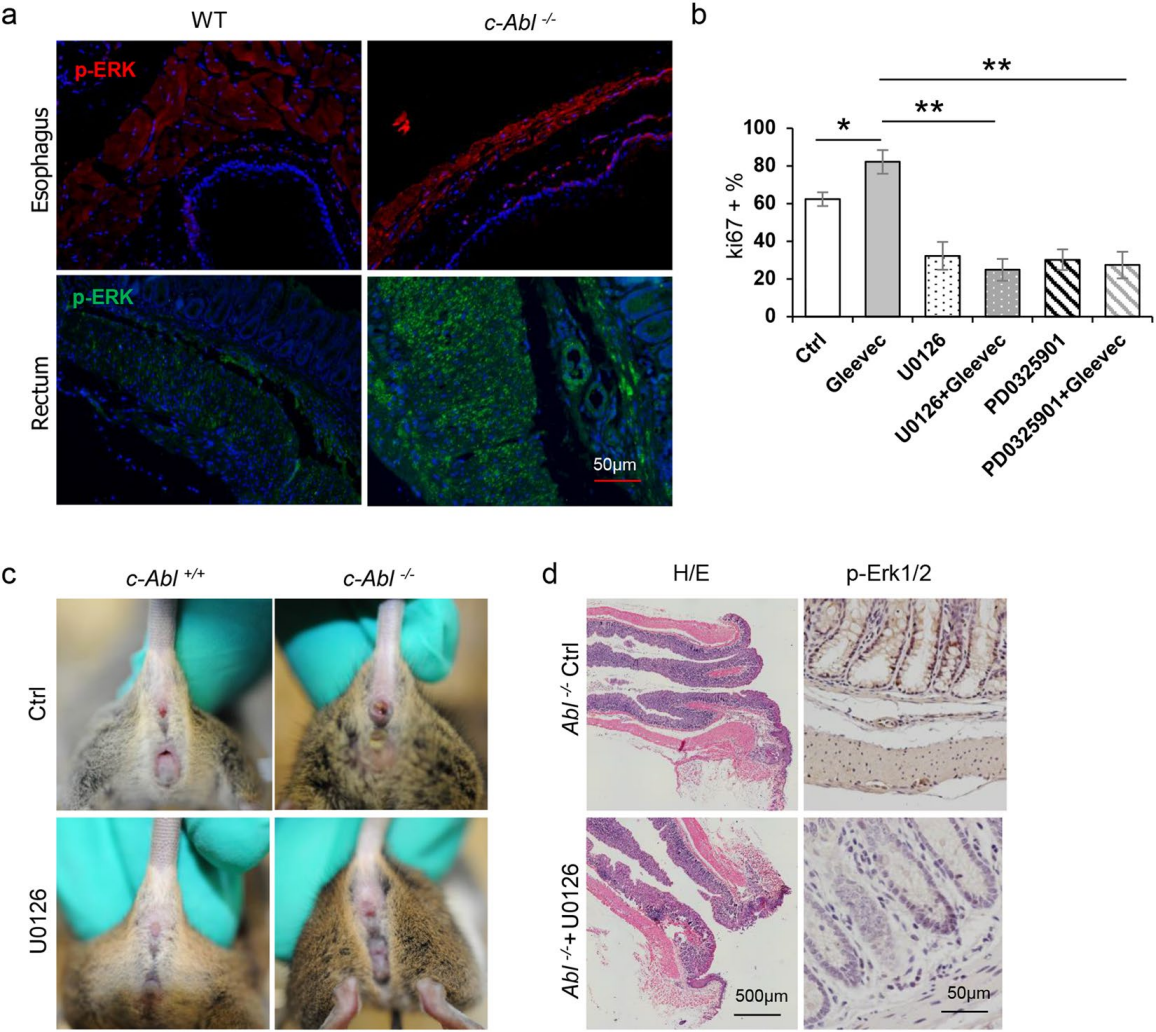
c-Abl deficiency increases smooth muscle cell proliferation due to enhanced ERK1/2 activation. (**a**) *c*-*Abl* ^−/−^ mouse esophagus and rectal tissues showed an increase in ERK activation. Tissues sections were incubated with p-ERK antibodies at 4 °C overnight, which were than incubated with FITC or TRITC conjugated secondary antibodies for 1 hr at room temperature. N = 3. (**b**) Inhibition of ERK with 20 μM of U0126 or PD25901 impeded c-Abl inhibition-induced overproliferation of smooth muscle cells. p = 0.010 (Ctrl and Gleevec), 6.5E-8 (Gleevec and U0126 + Gleevec), 2.0E-6 (Gleevec and PD0325901 + Gleevec), *p < 0.05 when compared to control. **p < 0.01 when compared to imatinib mesylate-treated cells. N = 3. (**c**) Administration of U0126 could alleviate rectal prolapse phenotype of *c*-*Abl* ^−/−^ mice. Representative picture of rectal prolapse in *c*-*Abl* ^−/−^ mice receiving U0126. N = 3. (**d**) Representative histological sections of the anus of *c*-*Abl* ^−/−^ mice receiving U0126. The organs were paraffin-embedded and the section slides were stained with Hematoxylin and eosin. Right panels: U0126 inhibited the activation of ERKs on rectum sections. N = 3.

Smooth muscle overproliferation in the absence of c-Abl activity suggests that c-Abl has a negative effect on the mitogenic pathways. Consistently, we found that imatinib mesylate treatment led to enhanced ERK1/2 activation on rectum sections (Fig. 6a). Consistent with previous finding that imatinib mesylate treatment led to activation of ERK1/2 in osteoblasts^18^, imatinib mesylate also led to an increase in ERK activation in smooth muscle cells (data not shown). Functionally, inhibition of ERK1/2 activation with U0126 or PD25901 could impede imatinib mesylate-induced increase in smooth muscle cell proliferation (Fig. 6b). More importantly, long-term administration of U0126 could prevent the development of rectal prolapse but not megaesophagus (Fig. 6c and data not shown), which were confirmed by HE staining of the colorectum (Fig. 6d). These data, taken together, suggest that c-Abl deficiency or inhibition leads to smooth muscle cell overproliferation via activating ERK1/2, which contributes to thickening of the muscularis propria layer in the gut and the development of rectal prolapse.

## Discussion

The gastrointestinal tract is responsible for food digestion and absorption and about 10% of the population experience GI tract disease at some time in their lives, including intestinal immobility and rectal prolpase. However, the pathogenesis of these disorders is largely unknown^42^. Our present genetic studies indicate that non-receptor tyrosine kinase c-Abl plays an important role in the homeostasis of the muscularis propria of the GI tract. c-Abl deficiency or inhibition led to defects in muscularis propria homeostasis and mice with c-Abl ablated in the whole body or in Prx1+ cells develop megaesophagus and rectal prolapse. Megaesophagus can be a symptom of achalsia due to the loss of ganglion cells in Auerbach’s plexus, or a symptom of myasthenia gravis, a neuromuscular disease^27^, or following the infection of the parasite Trypanosoma Cruzi^27^, while rectal prolapse mainly affects elder women and is believed to be caused by the weakening of the ligaments or muscle that holds rectum in place. Our findings suggest that pathogenesis of rectal prolapse may involve overproliferation of rectal smooth muscle cells, which can be a target for prevention and treatment of this disorder.

We found in the present study that megaesophagus and rectal prolapse develop in a progressive manner, with older mice showing more severe phenotypes. This is in line with the previous findings that *c*-*Abl* ^−/−^ mice show premature aging-related phenotypes such as osteoporosis^16–18^. Two critical regulators of aging at the cell and the organism levels are p16INK4a and p53^43^. By analyzing *c*-*Abl* ^−/−^ *p16Ink4a* ^−/−^ mice, we found that deletion of p16INK4a was able to rescue some of the defects including reduced fertility and shortened lifespan of *c*-*Abl* ^−/−^ mice, as well as defects in bone formation^21^, suggesting that p16INK4a is an important downstream effector of c-Abl. However, p16INK4a deficiency showed no effect on c-Abl deficiency-caused megaesophagus and only a minor effect on rectal prolapse, suggesting that these two aging-related disorders may not involve aging-promoting gene *p16Ink4a*. This is in contrast to the pro-aging roles for p16INK4a in bone formation, pancreases, and other organs^21, 44–46^. One explanation for these organ-specific effects of p16 could be that the smooth muscle cells in adult mice may have limited potential to proliferate, a process that is inhibited by p16INK4a.

How does c-Abl regulate the homeostasis of muscularis propria and how does c-Abl deficiency cause megaesophagus and rectal prolapse? *c*-*Abl* ^−/−^ mice, like imatinib mesylate-treated mice, seem to have problems in the proliferation and alignment of smooth muscle cells in most of the digestive organs. Recent studies show that c-Abl plays roles in myogenic differentiation and myoblast fusion^39, 47, 48^, smooth muscle cell proliferation *in vitro* ^41, 49^, and cardiomyocyte proliferation^36^. We showed that c-Abl plays a negative role in smooth muscle cells proliferation. c-Abl deficiency causes rectal prolapse by enhancing smooth muscle cell proliferation via Erks. The reason why c-Abl deficiency fails to cause a significant phenotype in small intestines may be that the small intestine contains much fewer microbes than the rectum. It is known that c-Abl can be activated by bacterial products such as lipopolysaccharide (LPS)^14, 22^, which may affect smooth muscle proliferation via c-Abl. Development of megaesophagus is a complex process^9, 50, 51^, whether c-Abl deficiency-caused megaesophagus is solely mediated by smooth muscle needs further investigation.

Our cell-based studies with c-Abl knockdown and inhibition and *in vivo* studies revealed that the anti-proliferation function for c-Abl in smooth muscle cells could be mediated by enhanced ERK1/2 activation, as ERK inhibitor U0126 not only suppressed smooth muscle cell proliferation but also the development of rectal prolapse in c-Abl deficient mice. This study thus reveals a link between c-Abl and ERK activation in smooth muscle cells, which appears to underlie the pathogenesis of rectal prolapse caused by c-Abl deficiency. Our findings also suggest ERK inhibitors may be drug candidates for the treatment of rectal prolapse.

The present study provides important information regarding imatinib mesylate, a drug used to treat CML and other cancers^10, 11^. We found that imatinib mesylate administration, like c-Abl deficiency, results in anomaly in the muscle tissue of esophagus and other parts of the GI tract. Recent clinical studies have revealed that long term use of imatinib mesylate could alter bone remodeling^52^ and toxic myopathy in mice and congestive heart failure in human^53^. On the other hand, a recent study suggests that imatinib mesylate can also reduce necrosis, inflammation, and fibrosis in a Duchenne Meryon muscular dystrophy mouse model^54^. Our present study uncovered another potential adverse effect of imatinib mesylate on smooth muscle maintenance in the GI tract, which suggests that in imatinib mesylate-treated patients, GI tract, especially the muscularis propria, should be monitored.

In summary, our studies uncover an important role for non-receptor tyrosine kinase c-Abl in the homeostasis of the muscularis propria of the GI tract, and suggest that *c*-*Abl* ^−/−^ mouse is a model for megaesophagus and (atypical) rectal prolapse, which are likely caused by increased proliferation of smooth muscle cells. Moreover, this study also implies that imatinib mesylate might have an adverse effect on the maintenance of the GI muscularis propria in patients and that ERK inhibitors may be effective to treat rectal prolapse.

## Materials and Methods

### Mice

*c*-*Abl* ^−/−^ and *c*-*Abl* ^*f/f*^ mice were generated at S. P. Goff’s lab of Columbia University and *p16Ink4a* ^−/−^ mice were from R. DePinho, Harvard University, USA. Prx1-Cre and ROSA-tdTomato mice were purchased from The Jackson Laboratory. All mice were maintained at Shanghai Jiao Tong University. All animal experiments were approved in accordance with the University of Shanghai Jiao Tong’s institutional guidelines on animal welfare [SYXK(SH)2011-0112]. All experimental methods were performed in accordance with the approved guidelines.

### Tissue preparation and histological analysis

Mice with the age from 1 day to 5 months were used in this study. The whole digestive tract were dissected out and rinsed in cold PBS. They were directly frozen in liquid nitrogen for RNA or protein isolation, or for cryosections. Slides of 8 μm- thickness were sectioned, fixed overnight, equilibrated in 30% sucrose, and embedded in OCT. Sections were stained with hematoxylin and eosin. For western blot analysis of c-Abl expression, these tissue samples were thawed on ice, weighted, and homogenated.

### Imatinib mesylate treatment of mice

Two-month-old mice were weighed and imatinib mesylate (Cat. S1026, Sellecchem, dissolved in saline) was intraperitoneal (IP) injected every other day at the dose of 5 mg/kg. The same amount of saline was injected as control. After 14 weeks, the mice were weighed and sacrificed. The whole GI tract was dissected out and fixed for histological analysis.

### RNA isolation and real-time PCR

Total RNAs were collected from esophagus of 3 pairs of mice using TRIzol (Invitrogen) following the product manual. RNA was subjected to reverse transcription following the manufacturer’s instructions (Roche). A portion of the reaction was used in real-time PCR assays.

### Cell culture and cell proliferation assay

Primary skeletal muscle cells were prepared from newborn mice, whereas primary mouse esophagus smooth muscle cells were purchased from Pricells (Shanghai, China). Log phase cells were treated with imatinib mesylate or transfected with c-Abl siRNA for three days. Trypan blue staining was used to count the cells every day.

### Immunofluorescence and Immunohistochemistry

Sections were blocked for 1hr at room temperature in 10% goat serum and 0.1% Triton. Sections were then incubated with anti-c-Abl (Santa Cruz, sc-887, 1:100), p-ERK antibodies (CST, 9106S,1:200), Ki67 (Abcam, ab15580, 1:150), CD3 (Abcam, ab16669, 1:100), αSMA (Sigma, A5228, 1:200) overnight at 4 °C, and incubated with secondary antibodies (Life, A-11001/A-11034, 1:100) for 30 min at 37 °C for Immunofluorescence staining, or incubated with Streptavidin Biotin Complex kit (BOSTER, SA1050) for immunohistochemistry staining.

### Western blot analysis

Tissues or cultured cells were lysed with TNEN buffer (50mM Tris, 150mM NaCl, 5 mM EDTA, 0.5% NP-40, 0.1% Triton X-100, 1 mM Na2VO3, 1 mM PMSF, 1 μg/ml aprotonin, 1 μg/ml leupeptin, 1 μg/ml pepstatin). The protein lysates of each sample was measured by BCA protein assay kit. Equal amounts of total proteins were loaded into 7.5% SDS-PAGE gel and transferred onto nitrocellulose membranes. Blots were incubated with the specific primary antibodies overnight and followed by incubating with secondary antibody and visualized by chemiluminescence. The western blot results were collected using a CCD camera and quantitated using the software provided by FluorChem M system (Protein Simple FM0405). We used Photoshop to process the images.

### Statistical analysis

Statistical analysis was performed using two-side Student’s test. P < 0.05 is considered a significant difference.

## Electronic supplementary material

Supplementary information
