# Supplementary material for: c-Abl regulates gastrointestinal muscularis propria homeostasis via ERKs
Source: Sci Rep. 2017 Jun 15;7:3563. doi: 10.1038/s41598-017-03569-0 (PMC5472598; doi:10.1038/s41598-017-03569-0)
Supplement: Supplementary file 1 — Supplementary information [file 41598_2017_3569_MOESM1_ESM.pdf]

Supplementary information

c-Abl regulates gastrointestinal muscularis propria homeostasis via ERKs

Jinnan Xiang[1]*, Yiqun Zhang[2]*, Dandan Bao[1], Na Cao[1], Xin Zhang[1], Ping Li[1], Shoutao Qiu[1], Jigang Guo[1], Dan He[1], Baojie Li[1], Liqing Yao[2,3], and Huijuan Liu[1,3]

Supplementary Methods

Primary skeletal muscle cell isolation

The skeletal muscle cell were excised form the hind limbs of postnatal 5-day-old mice, muscle tissues were cut into pieces and digested with typeIcollagenase and caspase at 37℃ for 30 min. Single cell suspension was obtained using a 70 µm strainer (BD Falcon). The cells were then collected via centrifugation and cultured in F10 medium supplemented with 20% fetal bovine serum.

Primary rectal muscle cells isolation

2-week-old mice were sacrificed and the rectum was separated, placed in a sterile dish, and washed with cold PBS plus PS. The rectum was cut along the longitudinal axis under a microscope, and the mucosal layer, submucosal layer and fascia layer were removed with forceps. The remaining transparent smooth muscle was transferred to the test tubes with PBS. The tissues were cut into pieces, and incubated with 100 U/ml type I collagenase at 37℃ for 30 min. The isolated cells and fragments were cultured in DMEM supplemented with 10% fetal bovine serum.

Ganglia staining

The colon were washed with PBS and fixed with 4% paraformaldehyde for 5h at 4℃. The tissues were cleared and opened, the mucous membrane, submucosa, and circular muscle were separated. After that, the remaining gastrointestinal myenteric plexus and longitudinal muscle were incubated in nicotinamide adenine dinucleotide phosphate (NADPH) diaphorase (NADPHd) (0.5 mg/ml nitroblue tetrozolium (Sigma), 0.3% Triton X-100, and 1 mg/ml NADPH (Sigma) in PBS) at 37℃ for 30 min. The pictures were taken under the Nikon stereomicroscope. The esophagus were embedded in OCT, sectioned of 50 µm-thickness and stained for NADPHd, as described above.

Supplementary figures and figure legends

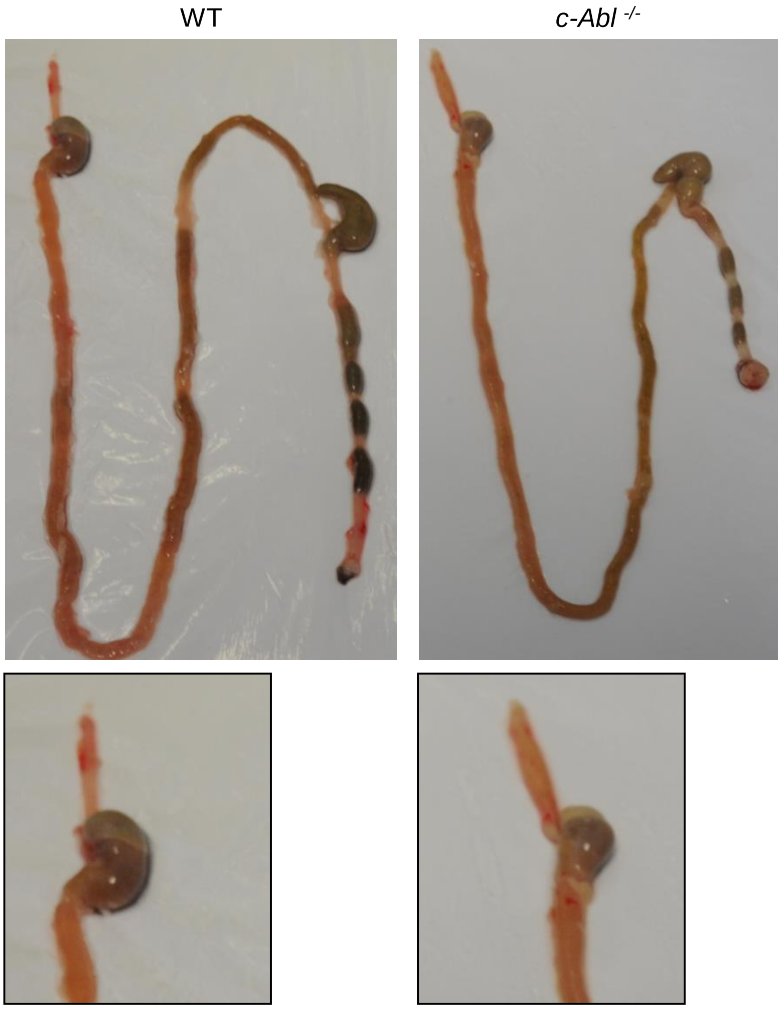

Figure S1. The whole GI tracts of a pair of wild type and *c-Abl*$^{-/-}$ mice. The GI tracts were dissected out and rinsed with cold PBS, from which the pictures were taken. N=5

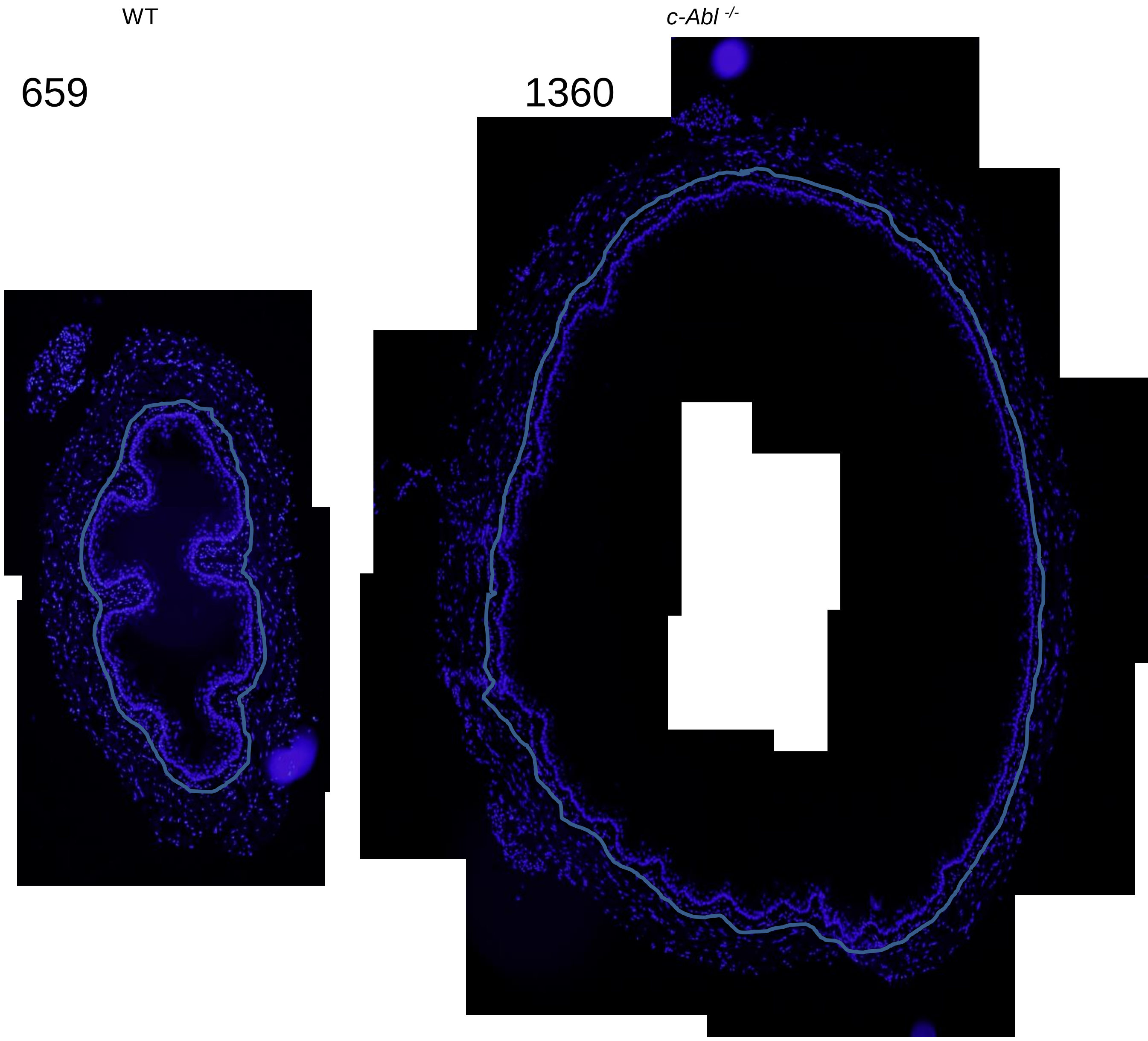

Figure S2. The number of smooth muscle cells in 5-month-old *c-Abl*$^{-/-}$ mice was increased compared to age-matched control mice. N=5

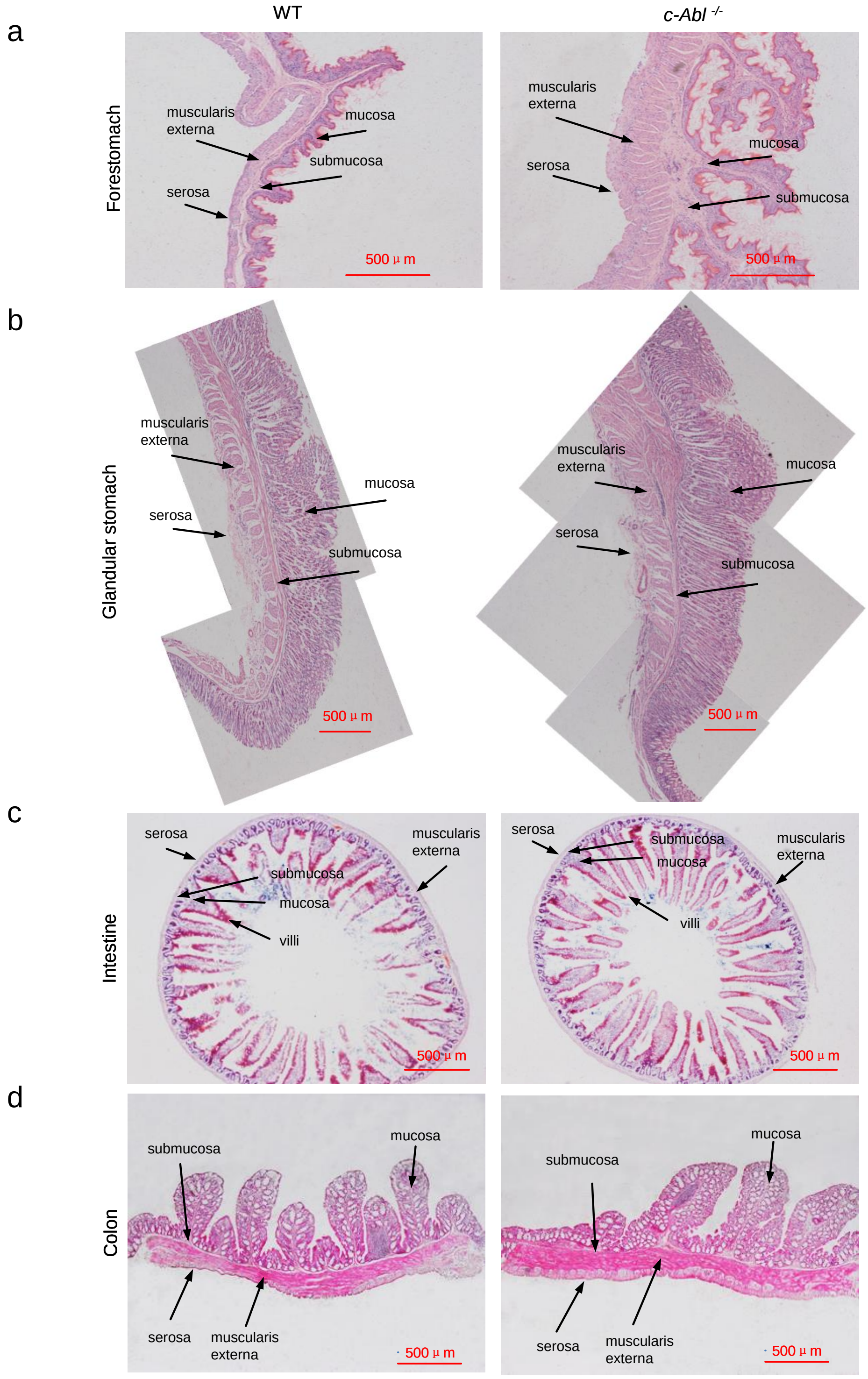

Figure S3. Muscularis propria phenotypes of the stomach, intestine and colon of *c-Abl*$^{-/-}$ mice.

a. Representative histological sections of the forestomach of *c-Abl*$^{-/-}$ and wild type mice. The organs were paraffin embedded and the section slides were stained with Hematoxylin and eosin. N=3

b. Representative histological sections of the glandular stomach of *c-Abl*$^{-/-}$ and wild type mice. The organs were paraffin-embedded and the section slides were stained with Hematoxylin and eosin. N=3

c. Representative histological sections of the small intestine (duodenum) of *c-Abl*$^{-/-}$ and wild type mice. The organs were paraffin-embedded and the section slides were stained with Hematoxylin and eosin. N=3

d. Representative histological sections of the colon of *c-Abl*$^{-/-}$ and wild type mice. The organs were paraffin-embedded and the section slides were stained with hematoxylin and eosin. N=3

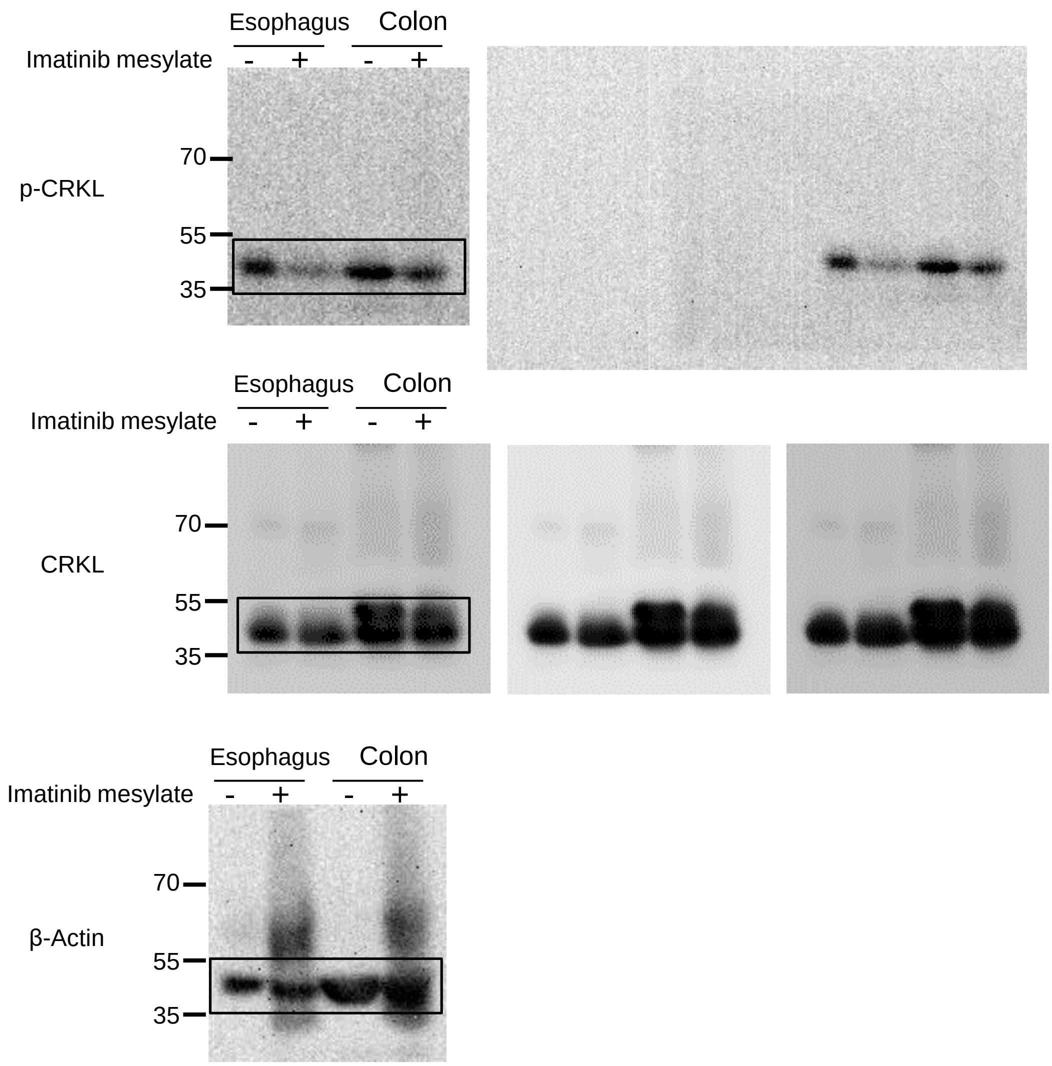

Figure S4. The unprocessed image of Fig 3A. N=3

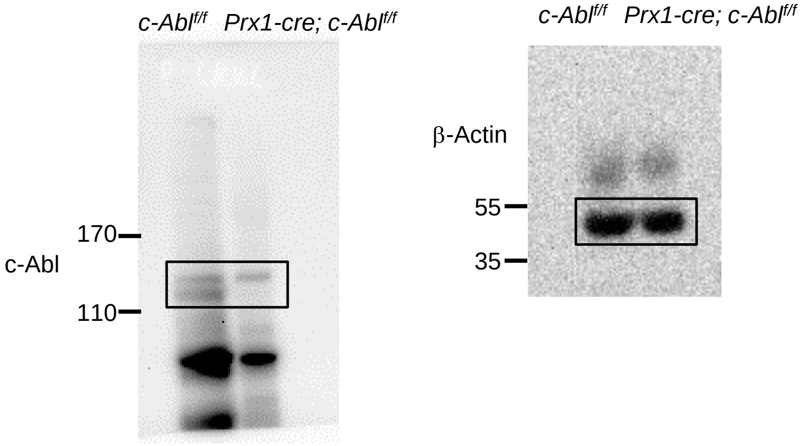

Figure S5. The unprocessed image of Fig 4B. N=3

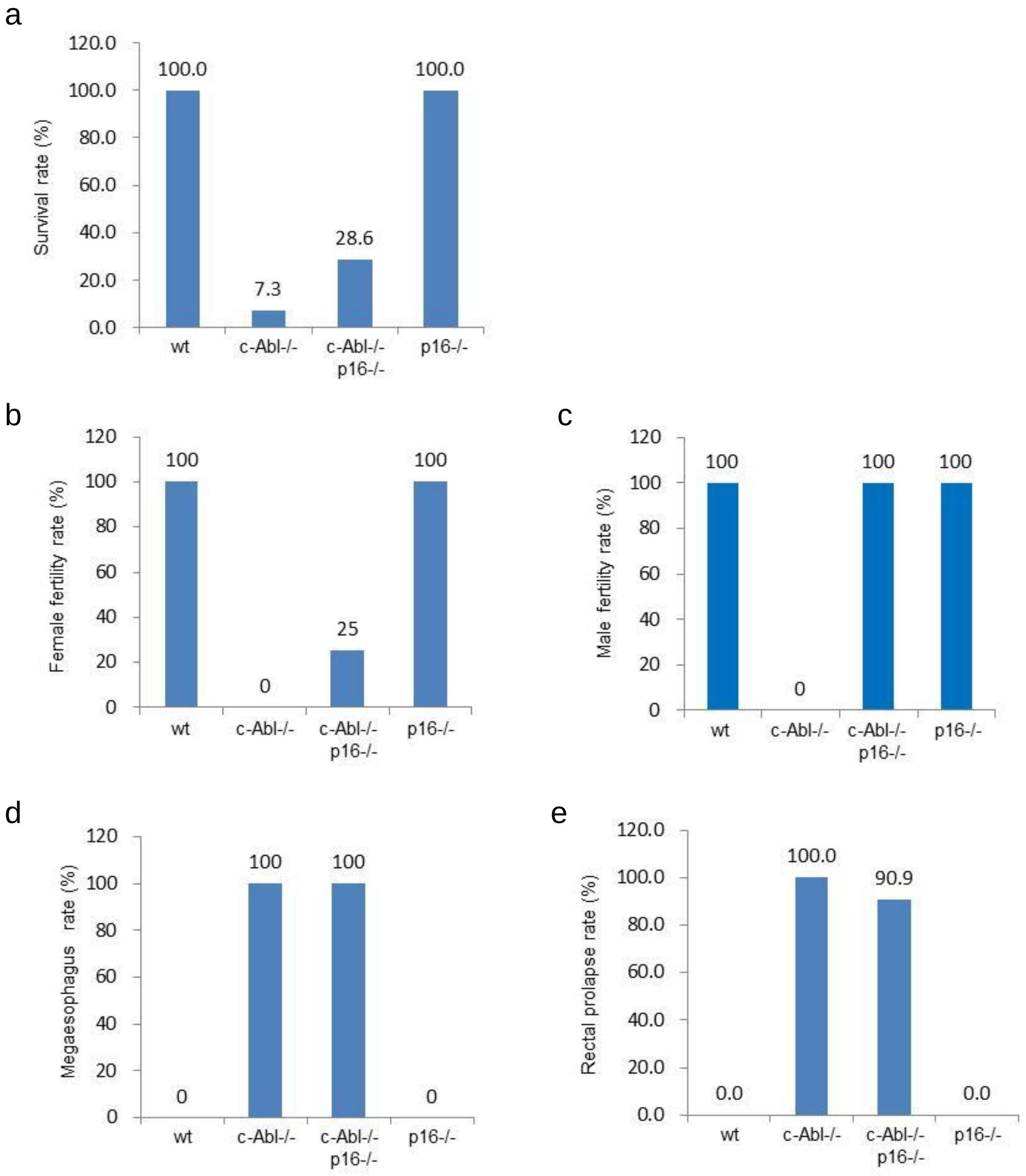

Figure S6. p16INK4a deficiency rescued some of defects of *c-Abl*$^{-/-}$ mice but not megaesophagus.

a. *c-Abl*$^{-/-}$*p16Ink4a*$^{-/-}$ mice showed an increase in postnatal survival rate compared to wild type mice. N=150

b. *c-Abl*$^{-/-}$*p16Ink4a*$^{-/-}$ female mice showed an increase in fertility compared to wild type mice. Mutant female mice were crossed to normal male mice and female mice that could give rise to offspring were deemed fertile. N=150

c. *c-Abl*$^{-/-}$*p16Ink4a*$^{-/-}$ male mice showed an increase in fertility compared to wild type mice. Mutant male mice were crossed to normal female mice and male mice that could give rise to offspring were deemed fertile. N=150

d. *c-Abl*$^{-/-}$*p16Ink4a*$^{-/-}$ mice showed a similar incidence of megaesophagus as *c-Abl*$^{-/-}$ mice. The number of mice with megaesophagus was normalized to the total number of mice. N=150

e. *c-Abl*$^{-/-}$*p16Ink4a*$^{-/-}$ mice showed a slight decrease in the incidence of rectal prolapse compared to wild type mice. The number of mice with rectal prolapse was normalized to the total number of mice. N=150

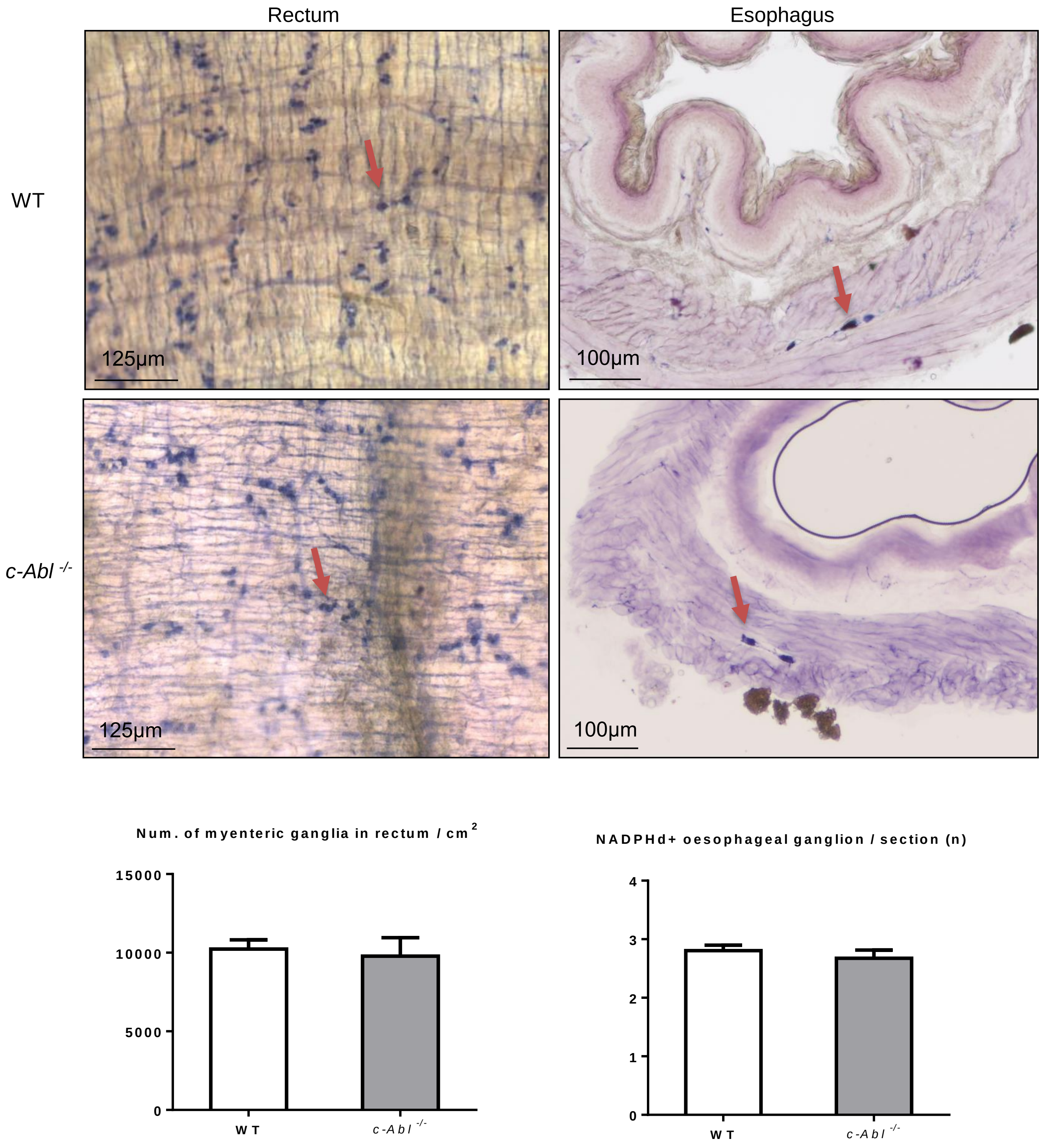

Figure S7. c-Abl deficient mouse esophagus and rectum did not show any difference in the number of ganglia cells. Bottom panels, quantitation data. The number of esophagus ganglia cells is on the entire cross-section of WT and mutant esophagus. n=50. Arrows indicate the ganglia cells. N=3

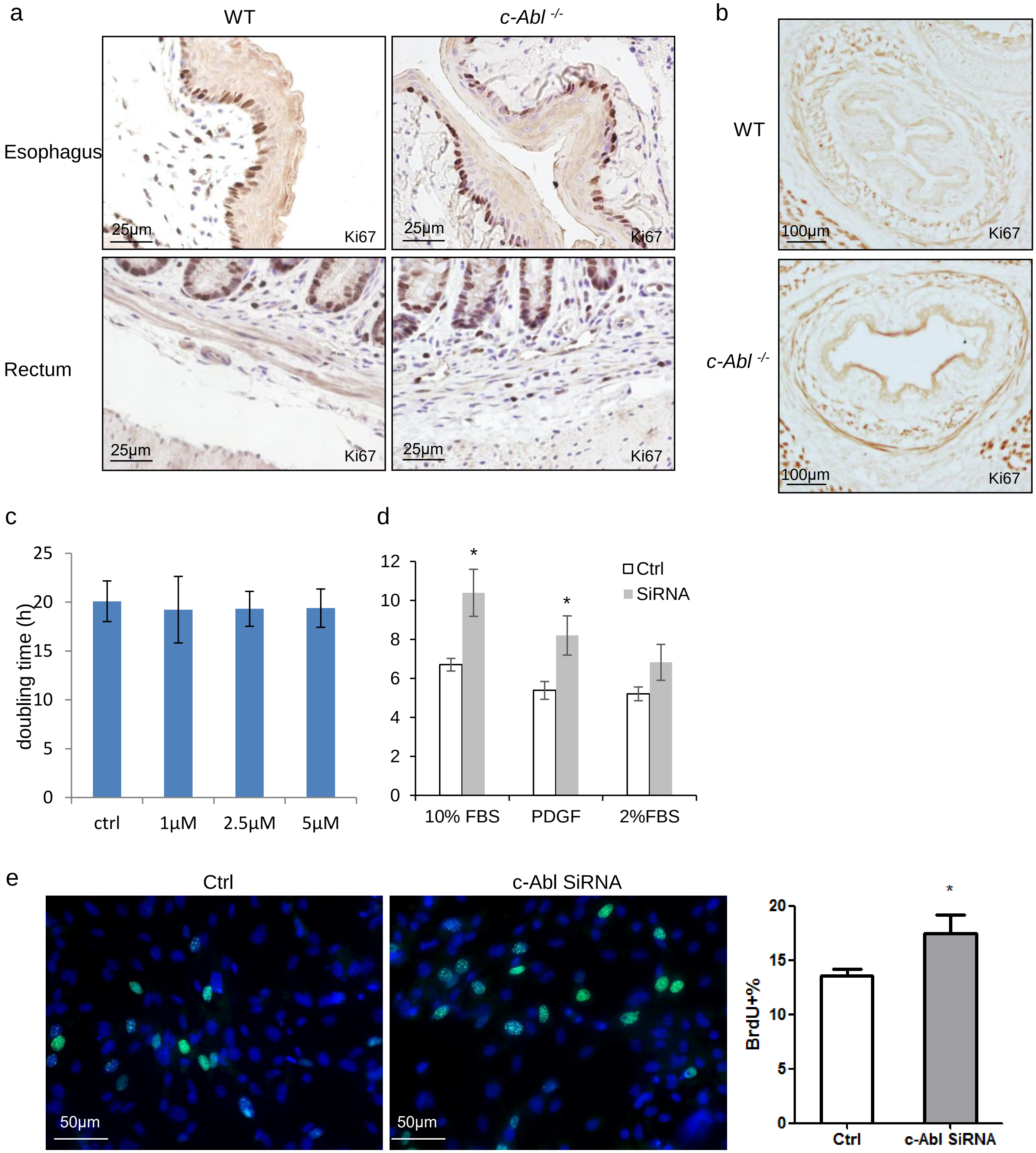

Figure S8. The effect of imatinib mesylate and c-Abl deficiency or knockdown on cell proliferation.

a. *c-Abl*$^{-/-}$ mouse esophagus showed a similar number of Ki67 positive cells as WT mouse. N=3

b. c-Abl deficient mice esophagus and rectum showed a similar number of Ki67 positive cells as WT mouse. N=3

c. Imatinib mesylate showed no effect on proliferation of skeletal muscle cells.N=3

d. c-Abl deficiency led to an increase in BrdU-labeled S phase cells in response to PDGF-AA or serum.N=3

e. c-Abl deficiency in primary rectal smooth muscle cells showed an increase in the number of BrdU positive cells compared to Ctrl. Right panel: quantitation data. P=0.0339, * $p<0.05$ when compared to control siRNA. N=3

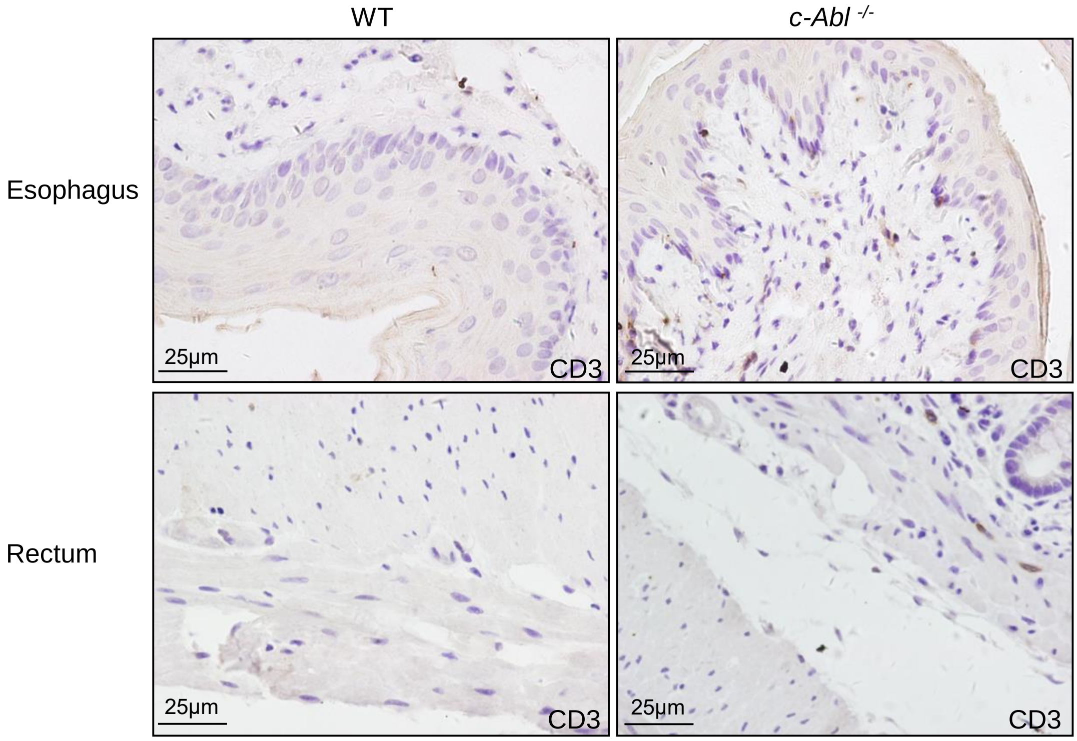

Figure S9. No difference of CD3 positive immune cells was observed in the esophagus and rectum between the *c-Abl* $^{-/-}$ and control mice. N=3

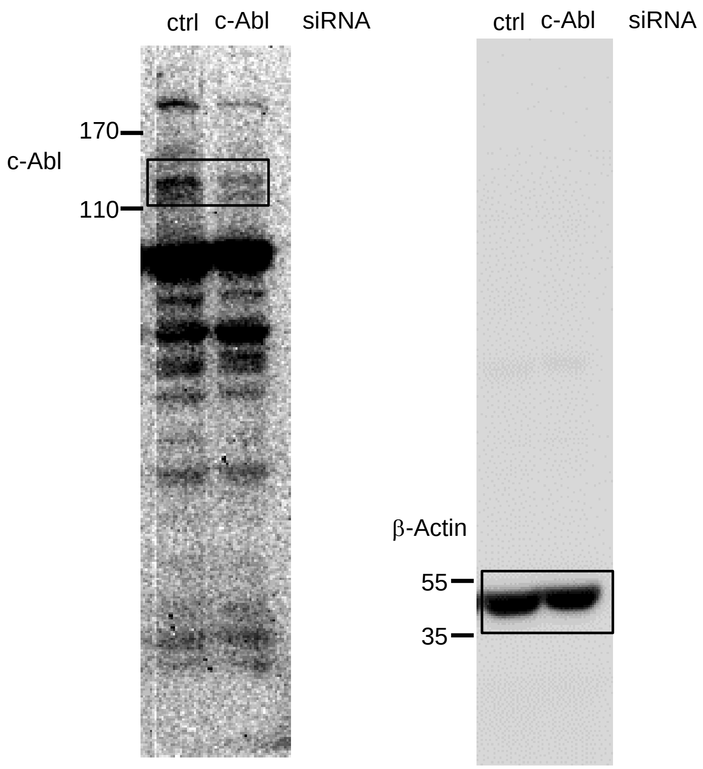

Figure S10. The unprocessed image of Fig 5C. N=5
